# When does social support help? Differential effects on the relationship between post-migration stress and internalizing symptoms in young male refugees and immigrant-origin peers from the Middle East

**DOI:** 10.1186/s13034-025-01003-2

**Published:** 2025-12-17

**Authors:** Usama EL-Awad, Arnold Lohaus, Tilman Reinelt

**Affiliations:** 1 Faculty of Psychology and Sports Sciences, Bielefeld University, P.O. Box 10 01 31, 33501 Bielefeld, Germany; 2 Faculty of Medicine, University Clinic of Psychiatry and Psychotherapy Luebbecke, Ruhr University Bochum, Bochum, Germany; 3 University Hospital, University of Zurich, Frauenklinikstrasse 10, 8091 Zurich, Switzerland

**Keywords:** Perceived social support, Post-migration stress, Internalizing symptoms, Refugee youth, Immigrant youth, Buffering effect, Middle Eastern adolescents, Young adults

## Abstract

**Background:**

Perceived social support is considered a crucial protective factor for mental health, particularly among marginalized populations. This study investigated the association between perceived social support and internalizing symptoms (depression, anxiety) in young refugees and immigrant-origin peers from the Middle East. It further examined whether social support buffers the adverse impact of post-migration stress on internalizing symptoms.

**Methods:**

The sample consisted of 135 male adolescents and young adults living in Germany, including *n* = 75 young refugees (primarily from Syria and Afghanistan) and *n* = 60 immigrant-origin peers, all with a Middle Eastern background. Validated self-report instruments were used to assess perceived support from friends, family, and significant others, as well as post-migration stress and internalizing symptoms.

**Results:**

While higher perceived support was consistently associated with fewer internalizing symptoms across both groups, a buffering effect on post-migration stress was found among young immigrants for all support sources, but not for their refugee peers (three-way interaction: β = – 0.46, SE = 0.18, *p* = 0.012; conditional interaction effect for immigrants: β = – 0.04, SE = 0.01, *p* = 0.008; for refugees: β = 0.01, SE = 0.01, *p* = 0.448). Refugees reported significantly higher post-migration stress than immigrant-origin peers (*t*(131) = 5.11, *p* < 0.001) and perceived less support from friends (*t*(132) = – 3.29, *p* = 0.001) and significant others (*t*(133) = – 2.41, *p* = 0.017) but not from family (*t*(130) = – 1.88, *p* = 0.063).

**Conclusions:**

These findings suggest that, for young male Middle Eastern refugees, perceived social support alone may be insufficient to buffer post-migration stress, underscoring the importance of expanding structural and instrumental support systems in Germany.

## Background

The successful social integration of immigrants is seen by many Western countries as a key strategy for counteracting demographic change in the form of an aging and shrinking society—and thus for securing long-term prosperity [73]. In addition, the wars in the Middle East, and recently also in the Ukraine, have led to an increased number of refugees, many of them children [70]. Consequently, the proportion of children and young people with an immigrant-origin background (i.e., born outside the country or with at least one parent born outside the country of residence) is increasing. For example, in Germany, 43.1% of children and young people had an immigrant-origin background in 2023 [23]. Young people with roots in the Middle East make up a significant proportion of their age group, at over 15%—a figure that more than doubled in just eight years by 2017 [15].

A notable characteristic of young refugees from the Middle East is the disproportionately high number of males in this group. Since 2016, most young refugees from this region in Germany have been male, with females making up only 18.9% of 16- to 18-year-old asylum seekers in the most recent data [40]. The proportion of males is even higher among unaccompanied refugees, reaching 91% [20, 24]. This underscores the need for dedicated research and a deeper understanding of their specific challenges in order to develop effective support strategies that foster a smooth integration in educational institutions and local communities.

## Young refugees and immigrant-origin peers from the Middle East: commonalities and divergences in challenges and contexts

Distinguishing between refugee and immigrant-origin groups is conceptually challenging, as such categories insufficiently represent the multi-faceted legal, political, and social conditions shaping migration (see, e.g., [10], for an empirically grounded discussion of this complexity). However as distinguishing between refugee and immigrant-origin groups is common, empirically studies are needed to reveal how the structural conditions that sustain such categorical attribution shape the access to resources, adaptation, well-being, and mental health after migration. The right to asylum, for example, was not conceived either officially or legally as an instrument of migration policy in Germany, but as a protective mechanism for individuals facing persecution. In social reality, however, these boundaries are becoming increasingly blurred: many refugees—including those from the Middle East—are staying in Germany for the long term, integrating into society and the labor market, and thus making a de facto contribution to mitigating demographic challenges. Thus, young people with roots in the Middle East should not be regarded as a homogeneous group, since not all of them have a history of flight, but rather in light of their differing migration contexts: Young *refugee adolescents and young adults* (hereafter referred to as RA) from this region were forced to migrate due to war and violence and have potentially experienced traumatic events before, during, and after migration that can have long-term effects on their mental health [42]. In contrast, non-refugee *first- and second-generation immigrant adolescents* and *young adults* (hereafter referred to as FSIA) have mostly immigrated as part of family migration, often for economic reasons, or were already born in the host country of their parents [62]. The latter usually show a higher degree of socio-cultural adaptation due to more favorable migration conditions—an important prerequisite for social integration [63]. In contrast, young RAs are often among the most marginalized groups. Due to their more recent arrival in the host country and the associated burdens, such as an ongoing asylum procedure or an uncertain residence status, they are generally exposed to significant social, structural, and psychological stressors that make it difficult for them to participate in society [31]. Conversely, their FSIA peers were often born in the country or have already migrated at a very young age—essentially, they grew up in the country. As a result, they have been able to establish more stable social networks through school and local communities and face fewer language barriers [74].

While distinguishing between different types of migration is complex, RA and FSIA from the Middle East continue to share common stressors due to overlapping aspects of the migration process and their socio-cultural context [18]. As a result, post-migration stress manifests itself due to intercultural conflicts, discrimination, labor-related problems, poverty, social isolation, and loneliness [16, 17, 30, 43, 47, 61]. Several studies have shown that post-migration stress may explain a high proportion of mental health outcomes including internalizing symptoms such as depression and anxiety in RAs and FSIA peers [13, 29, 34, 36, 64]. Furthermore, some authors suggest that the effects of post-migration stress could be of long-term nature—especially if this type of psychological burden makes it difficult to successfully cope with important age-appropriate developmental tasks [45, 68]. This, in turn, could inhibit and counteract social integration. Thus, the question arises: What resources can support young RA and FSIA in coping with post-migration stress, promoting their social integration, and preventing mental health problems. A particular important protective factor could be social support [46, 48, 57].

## The significant role of mental health and perceived social support in the integration of young male refugees and immigrant-origin peers

Social support involves interpersonal exchanges between individuals [75]. When perceived as adequate, it serves as a coping resource—a function supported by decades of empirical research. Particularly in male adolescence, social support is assumed to be an essential resource [52]. It can directly provide protective effect especially against internalizing symptoms including depression and anxiety [71]. Depending on the source, social support can be effective on a socio-emotional level, for example, through consolation or distraction when it comes from family, friends, or another close caregiver [7]. It can help to gain a new perspective on a problem and thus motivate active coping, while in the long term, it can also promote the development and establishment of problem-solving strategies [21]. However, according to buffering hypothesis [9], the effectiveness of social support in mitigating stress depends on whether the support is delivered adequately by the provider and whether it is perceived by the recipient as appropriate, relevant to the problem, and genuinely helpful. Particularly in the context of forced migration, the protective function of social support may not be universal: young refugees and their FSIA peers may differ in how they perceive and experience support from friends, family, or a significant other. For example, family support may be experienced differently depending on the degree of familial strain or shifting roles following migration and trauma. In addition, disruptions in socialization due to forced migration may affect how young refugees interpret closeness or advice from friends or caregivers, particularly when cultural expectations around emotion and gender remain unclear or conflicting. For instance, seeking socio-emotional support from peers or a significant other may conflict with norms of masculinity or privacy among young male refugees who have experienced trauma or instability [60]. In contrast, FSIA peers may have more stable family structures and be more open to socio-emotional support, perceiving it as more helpful and less conditional. Regarding unaccompanied youth, however, evidence suggests that their social support network could be inadequate or less accessible compared to that of their accompanied peers [57], highlighting the importance of considering these differences in analyses.

Finally, the effectiveness of perceived social support may be limited by legal insecurity. For young refugees with an unresolved asylum status, uncertainty about their future can overshadow social relationships and reduce the buffering effect of support on symptoms of depression and anxiety. Therefore, the meaning and perceived benefit of social support may vary substantially between RA and FSIA.

## Aims of the present study

This study aimed to investigate social support perceptions from various sources, including friends, family, and a significant other, among (a) young male refugees from the Middle East who arrived in Germany, and (b) their non-refugee first- and second-generation immigrant peers from the same or a similar Middle Eastern cultural region. The study examined whether perceived social support is associated with lower levels of internalizing symptoms in the context of post-migration stress. The objectives were:

1. Does the perception of social support in general as well as from friends, family, and a significant other differ between refugee adolescents and young adults (RA) and their first- and second-generation immigrant-origin (FSIA) peers from the Middle East in Germany?
2. Is the overall perceived availability of social support associated with less internalizing symptoms in both RA and FSIA from the Middle East?
3. Is there a buffering effect of perceived social support on the relationship between post-migration stress and internalizing symptoms, and does this buffering effect differ between RA and FSIA?
4. If a significant buffering effect is found for overall social support perceptions, post-hoc analyses will explore whether specific sources (friends, family, or significant other) contribute differently to this effect in RA and FSIA.

## Methods

### Procedure

This study was conducted as part of a research project focusing on the mental health of newly arrived young male refugees from the Middle East and their non-refugee immigrant-origin peers with Middle Eastern roots as well as their native peers . Only the first two groups, and not the latter, were considered in this study. Cross-sectional data were collected between 2016 and 2019 in several major German cities, during or shortly after the period in which over one million refugees from the Middle East arrived in Germany within a relatively short timeframe [40]. Participants were recruited in schools, immigrant associations, and sports clubs for refugees, as well as through direct approaches. Data collection took place at a German research center in separate rooms at youth organizations.

A local ethics committee at the University of Bremen approved the study procedure and the materials used. Before enrollment, participants and their legal guardians were fully informed about the objectives and procedures of the study. Participation was only possible after written consent had been provided by both the young people and, if necessary, their legal guardians. To strengthen the trust of the young refugees, it was explicitly emphasized that the study was entirely independent of state institutions, such as the police or immigration authorities, and that all data would be anonymized and used solely for scientific purposes. The study was conducted in individual or group settings with sufficient distance between the individual participants. The participants completed various questionnaires and cognitive tasks. All the data collection instruments were previously translated and back-translated into Arabic and Farsi by interpreters from the same ethnic groups as the participants, if not already available in the relevant languages. Scales that were not available in German were also translated and back-translated for immigrant peers. During data collection, an Arabic and Farsi interpreter was present with the study coordinator in the room, staying in the background to assist with clarifying questions.

### Participants

A total of 135 young male adolescents and young adults (*M*_age_ = 18.27 years, *SD*_age_ = 1.72 years) participated in the study. Participants either had a refugee background (*n*_RA_ = 75) or an immigrant-origin background as first- or second-generation youth and young adults (*n*_FSIA_ = 60). Young refugees were mostly from Syria, Afghanistan, and Iran, with an average length of stay in the host country of approximately 3 years (*SD*_stay_ = 1.29 years). The FSIA sample consisted of adolescents and young adults who were either born in Germany to Middle Eastern families without mixed parentage (35% of the total sample), or who had themselves immigrated from Iran, Afghanistan, or other Arab countries. Neither they nor their families had experienced forced displacement. Since all FSIA born abroad had excellent written and spoken German skills, it can be assumed that they immigrated at a young age and are therefore more comparable to their peers born in Germany. In addition to applying predefined inclusion criteria during participant recruitment, the study coordinator also conducted a brief, in-person screening interview with each participant to verify whether they had a refugee background. In addition, all participants answered during the survey whether they had experienced war or armed violence, and in the case of refugees, they were asked to indicate their asylum status. Finally, participants from the RA group were asked to indicate whether they had fled their home country alone, with family members, or friends. Among RA, the majority came from Syria and Afghanistan, representing the composition of most countries of origin of refugees in Germany in this age group at the time of data collection [24]. Young refugees underwent significantly less years of education than FSIA, *Welch’s t*(130.17) = − 5.63, *p* < 0.001. In comparison with FSIA, a larger proportion of the RA group had previously been employed in their home countries. Just over half of the RA had been granted asylum, while the remainder held other types of residence status. Almost half of the RA group came to Germany unaccompanied by close family members (e.g., parents). Most of both RAs and FSIAs were Muslim. All sample characteristics are displayed in Table 1.

**Table 1:** Sample characteristics

Factors		Groups	
		RA (*n* = 75)	FSIA (*n* = 60)
Age (in years), *M* (*SD*)		18.16 (1.64)	18.40 (1.80)
Country of origin, *n* (*%*)			
Syria		48 (64.0)	2 (3.3)
Afghanistan		17 (22.7)	10 (16.7)
Iran		7 (9.3)	11 (18.3)
Iraq		3 (4)	3 (5)
Germany		0	21 (35)
Other Arabic countries (e.g., Jordan)		0	13 (21.7)
Education in host and origin country (school years), *M* (*SD*)		9,0 (2.3)	10.9 (1.5)
Formerly employment, *n* (*%*)		32 (42.7)	19 (31.7)
Asylum status, *n* (*%*)			
Seeking (not yet registered)		18 (24)	
Applied		12 (16)	
Granted		42 (56)	
Rejected (tolerated stay)		3 (4)	
Unaccompanied status, *n* (%)		37 (49.3)	
Religion			
Islam		63 (84)	39 (65)
Christianity		7 (9.3)	5 (8.3)
Atheism/Agnothicism		5 (6.7)	16 (26.7)
Others		0	0

*Note*. RA = refugee adolescents and young adults; FSIA = first-and second-generation immigrant-origin peers

## Measures

### Perceived social support

The Multidimensional Scale of Perceived Social Support [75] is a widely used and well-validated instrument for assessing perceived social support with a socio-emotional focus (e.g., “I get the emotional help and support I need from my family.”). It has been validated with Arabic- and Farsi-speaking samples (e.g., [3, 41]). The scale consists of 12 items answered on a 7-point Likert scale, which are equally distributed across three subscales: family (Cronbach’s α = 0.84), friends (Cronbach’s α = 0.86), and significant others (Cronbach’s α = 0.87). Mean scores for each subscale and for overall perceived social support (Cronbach’s α = 0.95) were calculated with higher scores indicating higher levels of social support.

### Post-migration stress

The *Post-Migration Living Difficulties Scale* (PMLD; [58]) was adapted to the refugee and migration context of young Middle Eastern individuals in Germany. Participants were asked to rate the extent to which they had experienced common migration-related difficulties over the past 12 months (e.g., “Discrimination”) using a 5-point scale. The scale has demonstrated validity in studies with Arabic-speaking refugees and immigrants [32, 65] and has also been used with both first- and second-generation immigrant populations [72]. Since the original scale was primarily developed focusing on adult asylum seekers and refugees, certain items were removed because they did not reflect shared experiences between the RA and FSIA groups and were therefore not suitable for comparative analysis (e.g., “Interview with immigration”). The final version consists of seven items covering the following areas of difficulty: (1) discrimination, (2) inability to find work, (3) poor working conditions, (4) detention, (5) loneliness and boredom, (6) poverty, and (7) social isolation. A summative score of perceived post-migration stress was calculated (range: 7–35,Cronbach’s α = 0.88), allowing for a maximum of one missing item.

### Mental health

The *Hopkins Symptom Checklist-37A* (HSCL-37A; [4]) was used to assess internalizing symptoms over the past four weeks. Participants rated the frequency of these symptoms on a 4-point scale ranging from "never" to "always." The analysis focused on two subscales assessing depression (15 items and Cronbach’s α = 0.91) and anxiety (10 items,α = 0.87). To create a composite measure of internalizing symptoms, a summative score was calculated across all 25 items (range: 25–100), allowing for a maximum of one missing item in each subscale, and yielding high internal consistency (α = 0.94). For the German version, the *HSCL-25* [26] was used. The scale has previously been tested with Middle Eastern immigrants in Sweden [2, 67].

## Data preparation and preliminary tests

Data preparation and analysis were conducted with R 4.4.2 [49]. Missing values (3.2%) were managed at the scale level and treated using the Expectation Maximization (EM) algorithm [14]. A prior publication had already tested and confirmed the measurement invariance of the HSCL-37a and PMLD scale within the present dataset [18]. In addition, to test for possible differences concerning the PMLD scale within the FSIA sample between first- and second-generation immigrant participants, a Bayesian ANCOVA was conducted, including the PMLD total score as the dependent variable, the group variable (born in Germany vs. born abroad) as a predictor, and age as a covariate. The model with the group variable was not preferred over the model without this variable (BF₁₀ = 0.89), suggesting anecdotal evidence for the null model. The posterior estimate for the group effect was very small (β = − 0.11, 95% CI [− 1.83, 1.62], pd = 55%), suggesting that there is no meaningful or systematic difference between participants born in Germany and those born abroad in terms of their PMLD total score. In addition, a single Bayesian ordinal regression with each item was performed: All item-level models yielded BF₁₀ values below 1, providing anecdotal evidence in favor of the null model. Furthermore, prior to analysis, measurement invariance was tested using the lavaan R package (version 0.6–19; Rosseel, 2012) for the MSPSS scale between the RA and FSIA groups. To enable multigroup CFA with a relatively large set of indicators and to improve model stability and statistical power, item parcels were constructed for the MSPSS based on the item-to-construct balance method recommended by Little et al. [35]. Four parcels were created, each consisting of three items drawn evenly from the three original subscales (Family, Friends, Significant Other), based on the assumption of a unidimensional factor of perceived social support. This approach was chosen for pragmatic reasons to ensure analytical feasibility and because the study prioritized the total score of perceived social support in the main analyses and hypotheses. For instance, bifactor analyses (e.g.,[66]) have demonstrated that the general support factor can account for a substantial portion of reliably explained variance, suggesting that the total score may serve as a meaningful summary of perceived social support across sources. Measurement invariance was considered established when changes in both *CFI* and *RMSEA* remained within the range of *ΔCFI* ≤ 0.01 and *ΔRMSEA* ≤ 0.015 at the different invariance levels [8]. Testing the configural invariance for this scale resulted in fit measures of *CFI* = 0.987 and *RMSEA* = 0.054, and *ΔCFI* = -0.001 and *ΔRMSEA* = − 0.008 on the metric invariance level, *ΔCFI* = -0.009 and *ΔRMSEA* = 0.012 on the scalar invariance level. The results indicate that the MSPSS captures the same underlying construct across the groups.

## Analysis strategy

The RA and FSIA-group were assigned in the dataset using a binary variable. For the main analysis, all regression models controlled for age and unaccompanied status.

### Research Objective 1: Differences in perceived social support between RA and FSIA

Individual t-tests were calculated to examine differences between RA and FSIA with regard to perceived social support in the total scores on the one hand and in the individual sources (friends, family, significant other) on the other.

### Research Objective 2: Association between social support and internalizing symptoms

To test the general association between perceived social support and internalizing symptoms, a multiple regression analysis was conducted using the total social support score as predictor. A post-hoc analysis stratified by group was conducted to examine this association separately for RA and FSIA.

### Research Objective 3: Moderating effect of social support on the link between post-migration stress and internalizing symptoms among RA and FSIA

To test group-specific moderation effects, the model included a three-way interaction term (post-migration stress × social support × group variable), along with all associated two-way interactions (post-migration stress × social support, post-migration stress × group variable, and social support × group variable). This allowed testing whether the stress-buffering effect of perceived social support differed significantly between RA and FSIA. The moderating effects of post-migration stress were examined at three levels of perceived social support—low (16th percentile), mid (50th percentile), and high (84th percentile)—within each group.

### Research Objective 4: Contribution of individual social support sources

To explore the role of specific sources of perceived social support—if a significant effect was found for the total score—three separate moderated moderation models were estimated, one for each individual support source (friends, family, significant other). As in Research Objective 2, these models included the three-way interaction term and the lower-order interactions, to determine whether the buffering effect of each support source differed by group.

## Results

### Research Objective 1: Differences in perceived social support between RA and FSIA

Independent samples t-tests revealed that FSIA generally reported significantly higher levels of support (*M*_support total_ = 63.2, SD = 16.6) than RA (*M*_support total_ = 55.0, SD = 16.6), *t*(127.52) = – 2.80, *p* = 0.006, 95% *CI* [– 13.86, – 2.37]. In line with this, FSIA reported more support from significant others (*M*_*significant others*_ = 20.87, *SD* = 6.00) than RA (*M*_*significant others*_ = 18.15, *SD* = 7.10), *t*(133) = – 2.41, *p* = 0.017, 95% *CI* [– 4.95, – 0.49]. Support from friends was also significantly higher in FSIA (*M*_friends_ = 21.16, *SD* = 5.76) compared to RA (*M*_friends_ = 17.65, *SD* = 6.62), *t*(132) = – 3.29, *p* = 0.001, 95% *CI* [– 5.61, – 1.40]. However, the difference in family support between groups (in RA: *M*_family_ = 19.23, SD = 6.06; in FSIA: *M*_family_ = 21.13, *SD* = 5.63) was not statistically significant, *t*(130) = – 1.88, *p* = 0.063, 95% *CI* [– 3.89, 0.10].

### Research Objective 2: Association between perceived social support and internalizing symptoms

Levels of internalizing symptoms did not differ significantly between the two groups (in RA: *M*_internalizing symptoms_ = 44.25, *SD* = 15.40; in FSIA: *M*_internalizing symptoms_ = 42.41, *SD* = 12.34), *t*(133) = 0.77, *p* = 0.442, 95% *CI* [–2.88, 6.56]. Young refugee participants reported significantly higher perceived post-migration stress levels (*M*_post-migration stress_ = 20.40, *SD* = 7.50) than FSIA (*M*_post-migration stress_ = 14.08, *SD* = 6.83),* t*(131) = 5.11, *p* < 0.001, 95% *CI* [3.87, 8.76]. There was no significant difference in age between the two groups, *t*(119) = – 0.79, *p* = 0.431, 95% *CI* [– 0.84, 0.36].

Table 2 shows the correlations between internalizing symptoms, the perceived social support total as well as the individual source scores (family, friends, significant other), perceived post-migration stress, and age. Higher social support was related to lower internalizing symptoms across all subscales and the total score (Family: *r* = − 0.30, *p* = 0.006; Friends: *r* = − 0.28, *p* = 0.009; Significant Other: *r* = − 0.31, *p* = 0.003; Total: *r* = − 0.33, *p* = 0.002). Lower social support was in general associated with more perceived post-migration stress (*r* = − 0.23, *p* = 0.007), while the latter was positively associated with more internalizing symptoms (*r* = 0.36, *p* < 0.001). While correlations initially suggested that unaccompanied adolescents and young adults perceived less social support (*r* = − 0.18, *p* = 0.041), this effect was no longer significant when controlling for age (*p* = 0.706).

**Table 2:** Mean values, standard deviations, and correlations of the model variables

Variable	*M*	*SD*	1	2	3	4	5	6
1. Internalizing Symptoms	43.43	14.10						
2. Post-Migration Stress	17.59	7.84	0.36^***^					
3. SocSup Family	20.08	5.93	− 0.30^**^	-0.17				
4. SocSup Friends	19.21	6.47	− 0.28^**^	− 0.24^**^	0.74^**^			
5. SocSup Significant Others	19.36	6.75	− 0.31^**^	− 0.21^*^	0.69^**^	0.71^**^		
6. SocSup Total Score	58.64	17.21	− 0.33^**^	− 0.23^**^	0.89^**^	0.91^**^	0.90^**^	
7. Age	18.27	1.73	0.06	− 0.05	− 0.12	− 0.07	− 0.03	− 0.08

*Note. M* and *SD* represent the mean and standard deviation respectively. SocSup = perceived social support measure

* *p* < 0.05. ** *p* < 0.01, *** *p* < 0.001

### Research Objective 3: Moderating effect of social support on the link between post-migration stress and internalizing symptoms among RA and FSIA

Results of the moderated moderation analysis (see Table 3) indicate that higher post-migration stress was significantly associated with more internalizing symptoms. There was no significant difference in the average symptom burden between RA and FSIA. However, perceived social support showed a significant main effect, with higher levels of support being consistently associated with fewer internalizing symptoms, regardless of group membership.

**Table 3:** Moderated moderation analysis examining whether the effect of post-migration stress on internalizing symptoms via perceived social support differs between RA and FSIA groups

Predictor	β	*SE*^a^	*t*	*p*	LLCI^b^	ULCI^b^	
(Intercept)	− 0.11	0.10	− 1.13	0.262	− 0.31	0.09	
Post-migration stress	0.39	0.09	4.16	** < 0.001**	0.21	0.57	
SocSup TOT	− 0.34	0.08	− 4.06	** < 0.001**	− 0.51	− 0.17	
Group (refugees = reference vs. FSIA)	0.41	0.20	2.07	0.040	0.02	0.80	
Post-migration stress × SocSup TOT	− 0.12	0.08	− 1.35	0.018	− 0.29	0.05	
Post-migration stress × group	− 0.05	0.19	− 0.24	0.812	− 0.42	0.33	
SocSup TOT × group	− 0.11	0.17	− 0.63	0.529	− 0.45	0.23	
Post-migration stress × SocSup TOT × group	− 0.46	0.18	− 2.52	**0.012**	− 0.82	− 0.10	
Age	0.01	0.07	0.05	0.959	− 0.15	0.16	
Unaccompanied status (vs. others)	0.34	0.21	1.58	0.115	− 0.08	0.75	

Conditional effects of post-migration stress at values of SocSup and group							
SocSup (percentile)	Group						
Low (16th)	RA	0.32	0.16	1.90	0.059	− 0.01	0.65
Low (16th)	FSIA	0.72	0.25	2.85	**0.005**	0.22	0.99
Mid (50th)	RA	0.41	0.11	3.70	** < 0.001**	0.19	0.63
Mid (50th)	FSIA	0.30	0.14	2.16	**0.032**	0.02	0.59
High (84th)	RA	0.49	0.14	3.50	** < 0.001**	0.21	0.77
High (84th)	FSIA	− 0.01	0.15	− 0.11	0.904	− 0.31	0.28

*Note*. Model summary: *R*^2^ = 0.29, *F*(10,124) = 5.22, *p* < 0.001. RA = refugee adolescents and young adults; FSIA = first-and second-generation immigrant peers. SocSup TOT = total score value of perceived social support measurement. Test of interaction effect (Post-Migration Stress x SocSup) at group level: for RA, effect = 0.01, *F*(1, 124) = 0.57, *p* = 0.452; for FSIA, effect = -0.04, *F*(1, 124) = 5.96, *p* = 0.016. Percentiles based on total sample. Bolded p-values indicate statistical significance.

^a^ HC2-robust standard errors for heteroskedasticity-consistent estimation

^b^ 95% percentile bootstrap confidence intervals based on 5000 resamples

The three-way interaction indicated that the buffering effect of perceived social support on internalizing symptoms in the case of higher post-migration stress differed between RA and FSIA (β = − 0.05, *SE* = 0.02, 95% *CI* [− 0.09, − 0.01], *p* = 0.047). Separate follow-up analyses showed that the interaction between perceived social support and post-migration stress was significant for FSIA (β = − 0.04, *SE* = 0.01, *p* = 0.008), but not for RA (β = 0.01, *SE* = 0.01, *p* = 0.448). For FSIA, the association between post-migration stress on internalizing symptoms was significantly weaker at higher levels of perceived social support (e.g., at the 84th percentile—based on the total sample: β = − 0.01, *SE* = 0.15, *p* = 0.904), whereas in RA, the relationship remained positive across all levels of support (e.g., at the 84th percentile: β = 0.49, *SE* = 0.14, *p* < 0.001).

Post-hoc regression analyses which were carried out separately for RA and FSIA demonstrated that higher perceived social support was generally associated with fewer internalizing symptoms despite of age (or the unaccompanied status in case of RA)—in both RA (β = − 0.26, *SE* = 0.10, *p* = 0.017; *F*(10, 64) = 5.95, *p* < 0.001) and in FSIA (β = − 0.26, *SE* = 0.11, *p* = 0.031; *F*(9, 50) = 3.56, *p* = 0.012).

**Fig. 1:**
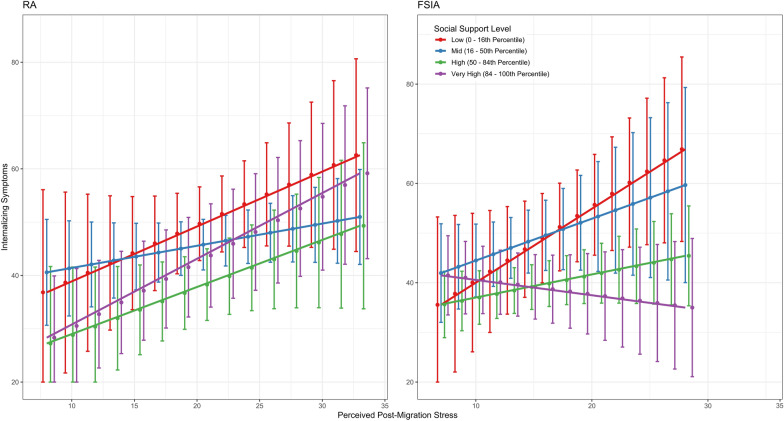
Perceived Post-migration stress and internalizing symptoms, moderated by social support levels in young refugees and immigrant-origin peers.* Note.* Perceived post-migration stress (sum scores) predicting internalizing symptoms (sum scores), moderated by social support in refugee adolescents and young adults (RA) and young immigrant-origin peers (FSIA). Lines = different levels of perceived socio-emotional support. Low: 0 – 16th percentile (of social support values); Mid: 16–50th percentile; High: 50–84th percentile; Very High: 84–100th percentile. Models were controlled for age, unaccompanied status, and country of origin

Figure 1 visualizes the conditional effects of perceived social support on the association between post-migration stress and internalizing symptoms. In the RA group, symptom levels generally increase across support levels. In contrast, FSIA peers indicate a divergence: While symptoms increase with low support, this relationship weakens at higher support levels, with the highest support group showing a nearly flat or slightly declining slope. Error bars illustrate substantial overlap in RA, suggesting limited differentiation, whereas greater uncertainty in FSIA—especially under high stress—reflects more variability in support effects.

### Research Objective 4: Contribution of individual social support sources

The results of the moderated moderation analysis for the individual sources of social support (see Table 4) showed that perceived support from family, friends, and significant others was generally associated with fewer internalizing symptoms, irrespective of group status (RA or FSIA). Moreover, the extent to which perceived social support buffered the association between post-migration stress and internalizing symptoms differed between RA and FSIA for all three sources of support.

**Table 4:** Moderated moderation analysis of the effect of post-migration stress on internalizing symptoms via perceived social support (individual sources: significant other, family, and friends)

Social support type/predictor		β	*SE*^a^	*t*	*p*	LLCI^b^	ULCI^b^
Family							
(Intercept)		− 0.12	0.10	− 1.19	0.233	− 0.32	0.07
Post-migration stress		0.35	0.09	3.86	** < 0.001**	0.17	0.53
SocSup		− 0.33	0.09	− 3.85	** < 0.001**	− 0.39	− 0.15
Group (refugees = reference vs. FSIA)		0.33	0.20	1.68	0.094	− 0.05	7.16
Post-migration stress × SocSup		− 0.06	0.09	− 0.64	0.521	− 0.23	0.12
Post-migration stress × Group		− 0.13	0.18	− 0.69	0.489	− 0.49	0.23
SocSup × group		− 0.13	0.17	− 0.79	0.428	− 0.47	0.20
Post-migration stress × SocSup × group		− 0.47	0.18	− 2.49	**0.013**	− 0.83	− 0.09
Age		0.01	0.08	0.10	0.917	− 0.15	0.17
Unaccompanied status (vs. others)		0.32	0.21	1.53	0.128	− 0.09	0.75
Friends							
(Intercept)		− 0.10	0.10	− 0.98	0.328	− 0.30	0.10
Post-Migration Stress		0.40	0.09	4.24	** < 0.001**	0.21	0.59
SocSup		-0.32	0.08	− 3.65	** < 0.001**	− 0.50	− 0.15
Group (refugees = reference vs. FSIA)		0.46	0.20	2.31	**0.022**	0.06	0.86
Post-migration stress × SocSup		− 0.15	0.09	− 1.62	0.106	− 0.34	0.03
Post-migration stress × Group		− 0.03	0.19	− 0.15	0.879	− 0.42	0.36
SocSup × group		− 0.31	0.18	− 1.70	0.090	− 0.67	0.04
Post-migration stress × SocSup × group		-0.46	0.19	− 2.38	**0.018**	− 0.87	− 0.08
Age		0.01	0.08	0.12	0.897	− 0.14	0.16
Unaccompanied Status (vs. Others)		0.37	0.21	1.75	0.081	− 0.04	0.80
Significant others							
(Intercept)		− 0.10	0.10	− 1.05	0.295	− 0.30	0.09
Post-migration stress		0.40	0.09	4.41	** < 0.001**	0.22	0.58
SocSup		− 0.31	0.08	− 3.73	** < 0.001**	− 0.48	− 0.14
Group (refugees = reference vs. FSIA)		0.38	0.19	1.93	0.055	− 0.01	0.76
Post-migration stress × SocSup		− 0.15	0.09	− 1.79	0.075	− 0.33	0.01
Post-migration stress × group		− 0.03	0.18	− 0.19	0.844	− 0.41	0.33
SocSup × group		− 0.08	0.17	− 0.47	0.634	− 0.43	0.26
Post-migration Stress × SocSup × group		− 0.48	0.18	− 2.59	**0.010**	− 0.85	− 0.11
Age		0.02	0.07	0.36	0.716	− 0.12	0.18
Unaccompanied status (vs. others)		0.29	0.21	1.38	0.167	− 0.16	0.71

*Note*. Model summary: For Significant Other, *R*^2^ = 0.28, *F*(9,125) = 8.87, *p* < 0.001. For Family, *R*^2^ = 0.27, *F*(9,125) = 7.58, *p* < 0.001. For Friends, *R*^2^ = 0.27, *F*(9,125) = 9.78, *p* < 0.001. RA = refugee adolescents and young adults; FSIA = first-and second-generation immigrant-origin peers. Test of interaction effect (Post-Migration Stress × SocSup) at group level: for RA, effect = 0.01, *F*(1,125) = 0.14, *p* = 0.712; for FSIA, effect = -0.11, *F*(1,125) = 9.63, *p* = 0.002 (Significant Other). For RA, effect = 0.05, *F*(1,125) = 0.79, *p* = 0.375; for FSIA, effect = -0.10, *F*(1,125) = 5.82, *p* = 0.017 (Family). For RA, effect = 0.02, *F*(1,125) = 0.09, *p* = 0.765; for FSIA, effect = -0.12, *F*(1,125) = 11.30*, p* = 0.001 (Friends). Bolded p-values indicate statistical significance.

^a^ HC2-robust standard errors for heteroskedasticity-consistent estimation

^b^ 95% percentile bootstrap confidence intervals based on 5,000 resamples

Further analyses in the individual groups showed that the buffering effect of the perceived social support sources on the association between post-migration stress and internalizing symptoms was significant in the case of FSIA, but not for RA. For FSIA, significant interaction effects were found for social support from family, friends, and significant others. In contrast, none of the sources showed a significant buffering effect for RA. The overall model fit was statistically significant for all three sources in both groups, for RA and FSIA.

## Discussion

Given the current public and political debates in Europe and beyond surrounding the integration of young male refugees and immigrants—particularly those from Middle Eastern countries—it is especially important to examine the protective role of social support in relation to mental health. This is not only due to its well-documented significance for mental health especially in resettlement contexts [56], but also because this group is often perceived more negatively than other ethnic groups in European societies and are exposed to a high level of discrimination [6, 54]. This may lead to restricted access to supportive social networks in host societies. This study investigated the impact of perceived social support from the immediate environment on the mental health of young males with Middle Eastern backgrounds living in Germany. The findings indicate that higher perceived social support—regardless of the source (friends, family, or significant others)—was generally associated with fewer symptoms of depression and anxiety. This direct effect was comparatively pronounced in both groups, although RA reported significantly less support than their FSIA peers. While FSIA experiencing high post-migration stress particularly benefited from social support and exhibited a clear buffering effect, this was not the case for their RA counterparts: In the RA sample, the relationship between perceived post-migration stress and internalizing symptoms was not moderated by the level of perceived social support. For their immigrant-origin peers in the FSIA sample, however, support from all three sources (family, friends, and significant others) had a stress-buffering effect.

## General beneficial and stress-buffering effects of perceived social support in young refugees and immigrant-origin peers

The significant main effect across the samples in this study shows that RA—just like their FSIA peers—generally benefit from perceived social support with regard to their mental health. This finding aligns with a substantial body of research on the protective role of social support [75]. However, the absence of a comparable buffering effect of support on post-migration stress in both groups suggests that additional contextual factors shape how social support is perceived and how it impacts mental health. Recently arrived young refugees are often at an early stage of the resettlement process, during which they have only limited access to social resources, which could help them navigate structural and societal challenges [59]. While FSIA are typically embedded in more stable social and legal frameworks, RA frequently face additional structural barriers—particularly when their residence status is unresolved, which can act as a long-term stressor. This legal uncertainty is often associated with significant daily and developmental limitations, such as restricted access to the labor market [5] or the healthcare system [33] and may indirectly contribute to further burdens like poverty [1], social isolation, and loneliness [51]. It is therefore conceivable that young refugees, due to the higher number of pervasive stressors that they face in daily life, do not feel that the social support available in their immediate surroundings is sufficient to regain a sense of control.

Moreover, an increasing number of studies indicate that experiencing early potentially traumatic stressors—such as war-related events including violence or assaults before or during the flight—are later linked to stronger perceptions of post-migration stress (e.g., [25, 36]). Due to this individual psychological and developmental vulnerability, it is possible that social support from significant people in their environment is not adequate on its own to mitigate the intensity of experienced stress without professional trauma-focused assistance. Although our analyses found that the unaccompanied status alone did not significantly explain the relationship between social support and internalizing symptoms compared to accompanied refugees, this may still be particularly relevant for young unaccompanied refugees. They are often exposed to high levels of stress and may be less likely to seek support due to unfamiliar surroundings, shame, or mistrust [11]. Sierau et al. [57] found that unaccompanied refugee minors without family contact perceived significantly less support from peers and mentors, and that limited support from these sources was associated with higher levels of PTSD, depression, and anxiety symptoms after stressful life events. In their cascade model of complex posttraumatic stress disorder, Maercker et al. [38] further highlight that early trauma in childhood and adolescence can lead to dysfunctional attachment patterns, which in turn may diminish the perception of social support later in life. Taken together, these findings suggest the need for more intensive, tailored support for young refugees. This leads to the stepped care approach, in which low-threshold interventions are offered in various facilities, with the option of transitioning to more intensive psychological treatment and therapy if necessary [37]. Preliminary results from the refuKey project in Germany with adult refugees from various countries of origin indicate an improvement in mental health when the stepped care principle is used, involving psychosocial counseling centers and psychiatric clinics at the regional level on an interdisciplinary basis [69]. However, no significant reduction in perceived stress after migration was observed, pointing to rigid and ongoing structural barriers. Hornfeck et al. [28] emphasize the feasibility and advantages of their stepped care approach in the reality of care for young unaccompanied refugees, focusing on a coordination between youth welfare services, psychotherapeutic care, and language teaching in Germany. At the same time, the authors draw attention to challenging circumstances regarding the degree of implementation between the participating institutions, which are partly due to staff uncertainty and limited confidence in dealing with mental health symptoms and behavioral problems. Hecker and Neuner [27] suggest a community-based stepped care approach in which trained peer counselors from refugee or immigrant communities actively approach newly arrived refugees of the same origin in their daily living environment (e.g., in accommodations) and offer brief screenings for psychological stress and trauma. Cases where the need for more intensive care is identified are treated by professional therapists in specialized outpatient clinics or trauma centers. If the severity of the symptoms requires further treatment, treatment facilities within the regular healthcare system are consulted. Stepped care models can therefore provide a promising framework for addressing the challenges associated with limited healthcare for young refugees in general.

## Differences in social support opportunities between refugees and immigrant-origin peers

While immediate social networks—such as family, friends, and significant others—typically offer socio-emotional support, this form of support primarily promotes affective well-being through internal psychological mechanisms [44]. The lack of a buffering effect of social support among RA could therefore suggest that emotional regulation alone is insufficient to counteract the effects of their post-migration stressors on mental health. Among FSIA, socio-emotional support may be more effective in buffering stress because it is often complemented by more stable access to instrumental resources, including financial, legal, and institutional support due to more established and resourceful family networks. Hence, in these family networks, social support may also include financial security or practical help—such as parental assistance in times of need—which may further enhance FSIA’s capacity to cope with stress. In contrast, for RA, the pervasive and often structural nature of the stressors they face—such as legal uncertainty, intersectional discrimination, or barriers to healthcare and education—may exceed what socio-emotional support from close contacts can compensate for.

Moreover, newly arrived refugees often rely on support from their own ethnic communities in the first years of their resettlement, which can be an important initial resource [19, 53] but may not provide the type of specialized assistance required to navigate complex administrative or legal challenges. This could reflect broader limitations in the capacity of RA’s personal networks to provide effective and targeted support [50]. Therefore, for this population, access to adequate professional legal guidance, psychosocial services, and structural assistance is not just beneficial, but essential.

## Limitations

The strength of the present study lies in the differentiated consideration of different migration contexts when examining perceived social support in the context of stress coping in young people with a Middle Eastern background in Germany. Furthermore, key covariates, such as the unaccompanied status of refugees, are taken into account in the analyses. Nevertheless, the study has some limitations: First, the cross-sectional study design does not allow any conclusions to be drawn about causal relationships or temporal changes in the effect of social support. Second, the transferability of the results to other refugee groups or host countries is limited, as they may live in a different structural, cultural and political context [55]. Third, the study was conducted exclusively with male participants, which raises the question of whether the findings can be transferred to female or non-binary refugees. Fourth, given the modest sample size, it is possible that smaller but still practically relevant effects were not detected, highlighting the need for replication in larger samples. Fifth, although measurement invariance was established for the total score of the MSPSS reflecting a general support factor, this study did not test measurement invariance for the three individual subscales due to limited sample size. However, prior research has demonstrated measurement invariance of the scale’s three-factor structure among Arabic-speaking populations (e.g., [22]). Sixth, the PMLD items used capture a subset of the post-migration stress experienced by both RA and FSIA participants. While an additional or expanded measure of stress could have provided more comprehensive insights, it may have also introduced unnecessary complexity and placed a greater strain on the young participants. Furthermore, objective stress measures (e.g., physiological indicators such as cortisol or heart-rate variability) often pose interpretational challenges in potentially traumatized populations, as they can be confounded with trauma-related physiological responses or general somatic distress and therefore do not necessarily reflect post-migration stress per se. These limitations should be addressed in future studies using longitudinal designs, larger and more diverse samples, and consideration of further influencing factors.

## Conclusions

This study showed that perceived social support is generally associated with better mental health, but that it does not always have a stress-buffering effect, especially for young refugees. Among young male immigrant-origin people with Middle Eastern background, those facing higher levels of post-migration stress appear to benefit particularly from social support, unlike their Middle Eastern refugee peers, for whom social support provides little relief regardless of stress intensity. Differences in the type of support–particularly the lack of instrumental help for refugees from professionals–could explain why social support from close persons alone is not enough to alleviate post-migration-related stress. The results underscore the need for more targeted institutional and societal measures to effectively relieve refugees through practical support in addition to socio-emotional support.

## Data Availability

No datasets were generated or analysed during the current study.

## Abbreviations

RA: Refugee adolescents and young adults
FSIA: First- and second-generation immigrant-origin peers
